# Frequent *KRAS* and *HRAS* mutations in squamous cell papillomas of the head and neck

**DOI:** 10.1002/cjp2.157

**Published:** 2020-01-20

**Authors:** Eiichi Sasaki, Katsuhiro Masago, Shiro Fujita, Nobuhiro Hanai, Yasushi Yatabe

**Affiliations:** ^1^ Department of Pathology and Molecular Diagnostics Aichi Cancer Center Hospital Nagoya Japan; ^2^ Department of Head and Neck Surgery Aichi Cancer Center Hospital Nagoya Japan

**Keywords:** squamous cell papilloma, *KRAS*, *HRAS*, HPV, head and neck

## Abstract

Squamous cell papilloma (SCP) is a benign neoplasm of the head and neck. Human papillomavirus (HPV) has been reported to be a tumourigenic factor for SCP. However, not all SCPs are positive for HPV, suggesting that other possible mechanisms are involved in their development. In this study, we examined the mutational status of 51 SCPs using targeted panel sequencing in addition to HPV status using GP5+/GP6+ PCR. HPV DNA was detected in 6 (12%) SCPs, while *KRAS* and *HRAS* mutations were detected in 18 (35%) and 17 (33%) SCPs, respectively. Notably, *KRAS* mutations, *HRAS* mutations and HPV infection were mutually exclusive. The larynx and trachea (4/7, 57%) were more preferentially infected by HPV than the other sites (2/44, 5%, *p* = 0.0019) and HPV was associated with multifocal development (4/5, 80%). In contrast, *KRAS* and *HRAS* mutations in SCPs were evenly distributed across the anatomical sites and found only in single SCPs. In conclusion, this study demonstrated that HPV was not frequently involved in SCPs and that *RAS* mutations were more common alterations. In contrast to inverted sinonasal papillomas and oncocytic sinonasal papillomas, SCP may not be a precursor lesion of carcinoma, because these aetiological events in SCP are distinct from squamous cell carcinoma in the same sites.

## Introduction

Squamous cell papilloma (SCP) is a relatively common benign lesion, showing papillary proliferation of squamous epithelium. SCPs develop widely in the mucosa of the upper aerodigestive system, including the oral cavity, pharynx, larynx, oesophagus, and trachea. The involvement of low‐risk human papillomavirus (HPV) in a subset of SCPs, particularly recurrent respiratory papillomatosis, is well known 1. However, the role of HPV in oral or pharyngeal SCPs appears to be limited 2, 3. These findings raise the question of whether HPV involvement might be different between single and multiple papillomas, and whether anatomical site might affect the association with HPV.

Consistent with the first study by Udager *et al* 4, we recently confirmed the specific involvement of *EGFR* mutations in inverted sinonasal papillomas 5. In a previous study, we also examined *KRAS* mutational status, resulting in the detection of the mutation in 38% of SCPs. Few reports have focused on oncogenic genetic alterations in SCPs. Therefore, we attempted in this study to address the relationship between HPV status and possible recurrent oncogenic drivers.

## Materials and methods

### Patients

We selected 51 SCPs of the head and neck (including 12 oesophageal papillomas) that were resected or biopsied in 51 patients from the database of the Department of Pathology and Molecular Diagnostics at Aichi Cancer Center Hospital, Nagoya, Japan. Twenty‐three of the SCP cases in this cohort were reported in a previous study 5. All diagnoses were confirmed by two experienced pathologists (ES and YY). All tissues were fixed in 10% formalin and embedded in paraffin. The study was approved by our Institutional Review Board.

### Mutation analysis

Tumour areas were marked on haematoxylin and eosin (H&E) stained sections. DNA was extracted from tumour areas on each unstained paraffin section while referring to the marks on the H&E‐stained sections. We confirmed that isolated tumour areas contained a minimum of 20% tumour cell nuclei. Targeted panel sequencing was performed on extracted DNA. These methods of detection have been described in detail elsewhere 6. In brief, sequencing libraries were generated from 10 ng of extracted DNA using a Hotspot Panel of 23 cancer‐related genes (see supplementary material, Table S1); variants were called using Ion Reporter 5.10 (Thermo Fisher Scientific, Waltham, MA, USA) and assessed using the CLC genomics workbench (Qiagen, Hilden, Germany).

### HPV analysis

HPV status was examined using GP5+/GP6+ consensus primers for the L1 region (150 bp product) 7, 8. We considered a sample to be HPV‐positive when GP5+/GP6+ PCR products were amplified and confirmed by direct sequencing using an ABI PRISM 310 Genetic Analyser (Applied Biosystems, Foster City, CA, USA). HPV types were determined using the NCBI Basic Local Alignment Search Tool 9. Additionally, we evaluated the presence or absence of koilocytosis in H&E‐stained specimens as koilocytosis is the morphological manifestation induced by HPV infection 10. Koilocytosis was diagnosed when epithelial cells contain an acentric, hyperchromatic, moderately enlarged nucleus with a large perinuclear vacuole, as described previously by Krawczyk *et al* 10.

### Statistical analysis

The chi‐squared test, the Fisher's exact test for independence, and the Kruskal–Wallis test were used to compare the frequencies of the clinicopathological variables. Statistical analysis was performed using StatView (version 5.0; SAS Institute Inc., Cary, NC, USA). A *P* value less than 0.05 was considered statistically significant.

## Results

### Patient characteristics

The patients were 40 men and 11 women with a median age of 63 years (range, 21–86 years). The majority (75%) had a history of smoking. The average tumour size was 5.4 mm (range, 2–20 mm). Five patients had multiple tumours; all five had synchronous multiple tumours, and two had local recurrences. None of the patients had malignant transformation and therefore there were no disease‐associated deaths.

### Genetic mutations and HPV status of SCPs

**Figure 1:**
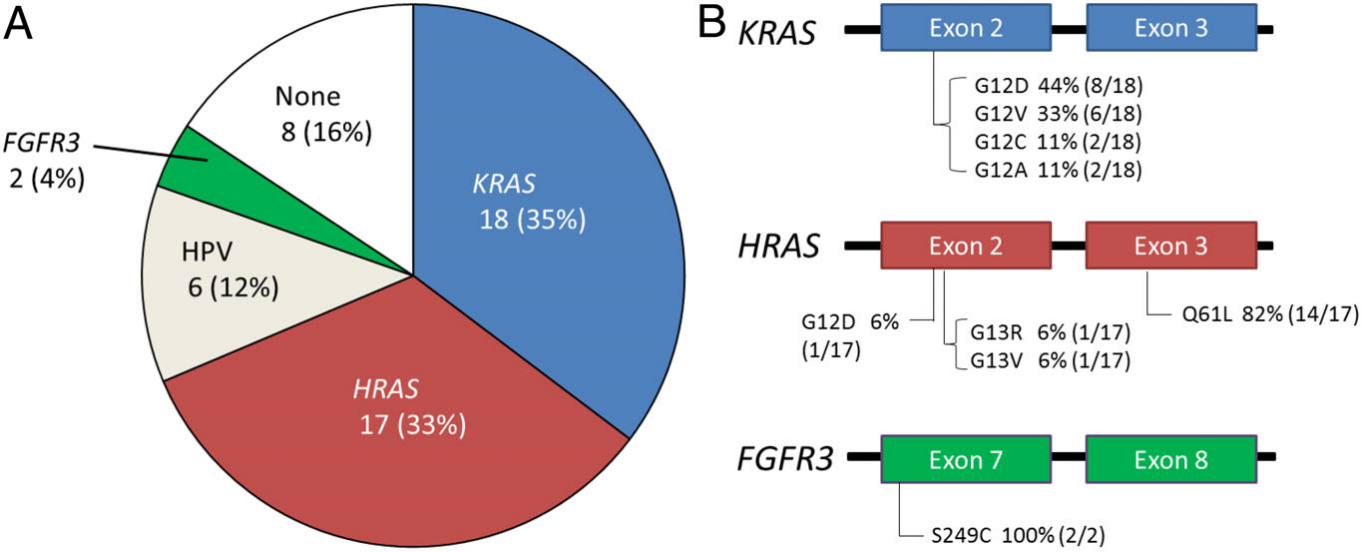
Molecular findings in SCPs. (A) Prevalence of *RAS*, *FGFR3* and HPV in SCP. HPV6 DNA was detected in five tumours, while the HPV type was unknown in the remaining tumour. (B) Distribution of *KRAS*, *HRAS*, and *FGFR3* mutations in SCP.

In a total of 51 SCPs from 51 patients, HPV DNA was detected in 12% (6/51) of tumours. The predominant virotype was HPV6, a low‐risk HPV type (5/6, 83%). *KRAS* and *HRAS* mutations were detected in 35% (18/51) and 33% (17/51) of tumours, respectively (Figure 1A) (see supplementary material, Tables S2 and S3). Four types of *KRAS* mutation, G12D, G12V, G12C and G12A, were found, while G12D, G13R, G13V, and Q61L were detected in *HRAS* (Figure 1B). Among the *RAS* mutations, *HRAS* Q61L was the most common variant (14/35, 40%), followed by *KRAS* G12D (8/35, 23%). In 10 of 51 (20%) tumours, neither *RAS* mutations nor HPV were detected. Interestingly, none of the tumours harboured two or more alterations of *RAS* mutations and/or HPV, suggesting a mutually exclusive nature of these alterations. In two tumours, *FGFR3* S249C mutations were detected, and both tumours were negative for *RAS* mutations and HPV infection.

### Clinicopathological characteristics of *RAS*‐mutated and HPV‐positive SCPs

Either *RAS* mutations or positive‐HPV were detected in 67% (8/12) of oral SCPs, 90% (18/20) of pharyngeal SCPs, 75% (9/12) of oesophageal SCPs, and 86% (6/7) of laryngeal/tracheal SCPs. The distributions of the alterations were significantly different according to the anatomic sites (*p* = 0.0026, Table 1). This significant difference was due to the high incidence of HPV infection in the larynx and trachea (4/7, 57%) and low incidence in the other sites (2/44, 5%) (larynx/trachea versus oral cavity, *p* = 0.0379; larynx/trachea versus pharynx, *p* = 0.0020; larynx/trachea versus oesophagus, *p* = 0.0379; Fisher's exact test). Moreover, the frequency of *KRAS* mutations was significantly higher in the pharynx (11/20, 55%) than in the oral cavity (1/12, 8.3%) (*p* = 0.0107). In contrast, there was no significant difference in the distribution of *HRAS* mutations according to the anatomic sites. It was also different between single and multiple occurrence; HPV‐positive SCPs were frequently multiple (4/6, 67%), while *RAS*‐mutated SCPs were always single (HPV‐positive SCPs versus *RAS*‐mutated SCPs, Fisher's exact test, *p* = 0.0001).

**Table 1:** Clinicopathological characteristics of squamous cell papilloma

Characteristics	Number/value/range	*KRAS*	*HRAS*	HPV	None	*P* value
Sex						0.7232
Male	40	14	13	4	9	
Female	11	4	4	2	1	
Age						0.8847
Range	21–86	21–81	44–76	39–74	34–86	
Median	63	59.5	64	58.5	63	
Smoking status*						0.7697
Current/former	33	13	13	2	5	
Non‐smoker	11	4	3	1	3	
Site						0.0026
Oral cavity	12	1	6	1	4	
Pharynx	20	11	7	0	2	
Oesophagus	12	4	4	1	3	
Larynx/trachea	7	2	0	4	1	
Single or multiple						<0.0001
Single	46	18	17	2	9	
Multiple	5	0	0	4	1	
Size (mm)						0.1995
Range	2–20	3–11	2–7	3–6	2–20	
Mean	5.4	5.6	4.2	4.0	7.9	
>5 mm	15	7	3	1	4	
≤5 mm	36	11	14	5	6	
Koilocytosis						<0.0001
Presence	4	0	0	4	0	
Absence	47	18	17	2	10	

* Some records were missing.

**Figure 2:**
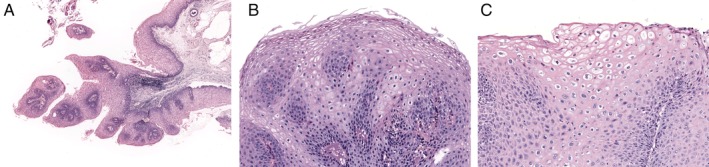
Histological findings of SCPs. (A) H&E image of an oral SCP with an *HRAS* mutation at ×40 original total magnification. (B) H&E image of a pharyngeal SCP with a *KRAS* mutation at ×200 original total magnification. (C) H&E image of an HPV‐positive laryngeal SCP with koilocytosis at ×200 original total magnification.

Histologically, koilocytosis was found in 67% (4/6) of HPV‐positive SCPs but absent in HPV‐negative SCPs (HPV‐positive SCPs versus HPV‐negative SCPs, Fisher's exact test, *p* < 0.0001) (Figure 2). The histological findings of SCPs were not different between *KRAS‐* and *HRAS‐*mutated SCPs. None of the tumours had dysplasia.

## Discussion

In this study, we found that HPV was associated with laryngeal/tracheal and multicentric development. In contrast, *RAS* mutations accounted for the major alterations (69%, 35/51) in the SCPs, and were mutually exclusive of HPV positivity in a manner independent of the anatomic sites.

The oncogenic mechanism of HPV infection has been shown to be related to the intrinsic proteins of HPV. The E5 protein of HPV activates the mitogen activated protein kinase (MAPK) pathways, a downstream target of *RAS* genes 11, 12. Therefore, *RAS* mutations and low‐risk HPV lead to activation of the same RAS‐MAPK pathway, suggesting a crucial role in molecular pathogenesis. Mutually exclusive involvement of HPV and *RAS* also supports the importance of this pathway.

Specific involvement of HPV in the larynx and trachea might be associated with the histological characteristics of the sites. Recurrent respiratory papilloma, a representative HPV‐related papilloma, tends to occur in the transition zone of the larynx between stratified squamous epithelium and ciliated epithelium 1, 13. This zone includes regional stem cells, which may be important for persistent infection by HPV. Indeed, HPV infection in the uterine cervix is first detected in transitional zone cells (squamocolumnar junction cells), and the infection is suggested to initiate neoplastic processes 14, 15. These findings suggest that the anatomic unit has different susceptibility to particular pathogenesis, such as HPV in squamocolumnar or transitional zone cells.

Our results first revealed that *KRAS* and *HRAS* mutations were the major genetic alterations in SCPs. These findings in SCPs are in line with those of previous studies. Frequent *KRAS* mutations are found in oncocytic sinonasal papilloma 16, 17, and urothelial papilloma 18, while *HRAS* mutations are frequent in inverted urothelial papilloma 18, 19. The involvement of the RAS pathway is also represented by mouse models. *Hras* and *Kras*, but not *Nras*, activation induces papillomas in the skin and oral mucosa 20, 21, 22, 23. Notably, the susceptibility associated with *Ras* mutations in mice is different among cancers; *Hras* induced papillomas and haematopoietic tumours, whereas *Kras* elicited gastric tumours and lung lesions 24. The divergent responses to *RAS* signal activation are explained by a narrow window of *RAS* mutations for tumourigenesis. Too little signalling leads cells to fail to proliferate, while too much signalling leads to abortive processes, such as growth arrest, that may result in a benign lesion incapable of further progression to carcinoma. In addition, the magnitude of the optimal signal varies according to the intrinsic cellular context 25. Therefore, individual sites may have a particular expression level of specific mutated genes to generate the tumour.

Interestingly, the mutational spectrum is distinct between the papillomas and carcinomas that arise from the same sites. *KRAS* mutations in head and neck cancers are extremely rare (less than 1%), whereas *HRAS* Q61L mutations, the most common variant in SCPs, are found in a small proportion 25, 26, 27. Actually, malignant transformation of SCPs is extremely rare 1, 28. These findings may be partially explained by the virotype of HPV; HPV in SCPs was restricted to low‐risk types, such as HPV 6 and 11. Furthermore, the different spectrum of *RAS* mutations supports the suggestion that papilloma is not a precursor lesion of squamous cell carcinoma of the head and neck. We speculate that features of the tumour are closely associated with the anatomical site, which defines susceptibility, and alterations, including the type of genes involved and the expression level of the mutated gene, according to the intrinsic cellular context.

There are several other types of papilloma in the head and neck. Malignant transformation has been reported to occur in 2–27% and 4–17% of cases of inverted sinonasal papilloma and oncocytic sinonasal papilloma, respectively 29, 30. The majority of cases of these carcinomas associated with papillomas are synchronous 4, 5. Recent studies have shown a genetic link between both components 4, 5, 16. Therefore, inverted sinonasal papilloma and oncocytic sinonasal papilloma are considered as precursor lesions of sinonasal cancer. In contrast, malignant transformation of exophytic sinonasal papilloma, like that of SCP, is extremely rare although exceptional cases have been reported 29, 31. Furthermore, consistent with a recent large study on SCP 32, no SCP showed dysplasia in our study. Taken together with the different mutational spectrum between SCP and cancer of the head and neck, it is difficult to consider that SCP is a precancerous lesion.

In this study, 20% (10/51) of all SCPs were negative for *RAS* mutations and HPV infection. *FGFR3* S249C mutations were detected in two SCPs without *RAS* mutations or HPV infection. In line with the previous discussion, this mutation can activate the MAPK pathway 33, further suggesting a crucial role for *RAS* activation in SCPs.

The association of SCP tumourigenesis with smoking has remained unknown. The mucosa in the upper aerodigestive tract is directly exposed to smoking, and head and neck cancer is closely related to smoking 34. Similarly, HPV infection is more frequent in smokers 35. Therefore, we also analysed the relationship with smoking status. However, there was no relation in this study between *RAS*/HPV status and smoking history irrespective of anatomic site (data not shown). However, this may be masked due to the high percentage (33/44, 75%) of patients with a history of smoking.

In conclusion, our study shows frequent involvement of the RAS‐MAPK pathway in SCPs, including *RAS* mutations and low‐risk HPV infection. These early aetiological events may have crucial implications for developing the benign nature of SCPs.

## Author contributions statement

ES and YY conceived and designed the study. ES, NH and YY contributed to the materials and patients. ES and YY provided pathology review. ES, KM and SF carried out experiments. ES, KM, SF and YY analysed and interpreted the data. ES and YY generated tables and figures. All authors were involved in writing the paper and had final approval of the submitted manuscript.

## Supporting information


**Table S1.** Hotspot panel of 23 cancer‐related genes

**Table S2.** Oncogenic mutations related to the MAPK pathway and HPV status for 51 SCPs

**Table S3.** Average read depth for the targeted *KRAS*, *HRAS*, and *FGFR3* amplicons
