# Supplementary material for: Frequent KRAS and HRAS mutations in squamous cell papillomas of the head and neck
Source: J Pathol Clin Res. 2020 Jan 20;6(2):154–9. doi: 10.1002/cjp2.157 (PMC7164371; doi:10.1002/cjp2.157)
Supplement: Supplementary file 1 — Table S1. Hotspot panel of 23 cancer‐related genes [file CJP2-6-154-s001.docx]

# Frequent *KRAS* and *HRAS* mutations in squamous cell papillomas of the head and neck

Sasaki E *et al. J Pathol Clin Res* DOI: 10.1002/cjp2.157

**Table S1. Hotspot panel of 23 cancer-related genes**

| *KRAS* | *AKT1* | *STK11* | *CTNNB1* | *FBXW7* | *FGFR1* |
| --- | --- | --- | --- | --- | --- |
| *EGFR* | *ERBB2* | *MAP2K1* | *MET* | *FGFR3* | *FGFR2* |
| *BRAF* | *PTEN* | *ALK* | *TP53* | *NOTCH1* | *HRAS* |
| *PIK3CA* | *NRAS* | *DDR2* | *SMAD4* | *ERBB4* |  |
